# The Protein Disulfide Isomerase of *Botrytis cinerea*: An ER Protein Involved in Protein Folding and Redox Homeostasis Influences NADPH Oxidase Signaling Processes

**DOI:** 10.3389/fmicb.2017.00960

**Published:** 2017-05-29

**Authors:** Robert Marschall, Paul Tudzynski

**Affiliations:** Institut für Biologie und Biotechnologie der Pflanzen, Westfälische Wilhelms-Universität MünsterMünster, Germany

**Keywords:** *Botrytis*, NADPH oxidase, protein folding, ER, virulence, signaling

## Abstract

*Botrytis cinerea* is a filamentous plant pathogen, which infects hundreds of plant species; within its lifestyle, the production of reactive oxygen species (ROS) and a balanced redox homeostasis are essential parameters. The pathogen is capable of coping with the plant’s oxidative burst and even produces its own ROS to enhance the plant’s oxidative burst. Highly conserved NADPH oxidase (Nox) complexes produce the reactive molecules. The membrane-associated complexes regulate a large variety of vegetative and pathogenic processes. Besides their commonly accepted function at the plasma membrane, recent studies reveal that Nox complexes are also active at the membrane of the endoplasmic reticulum. In this study, we identified the essential ER protein BcPdi1 as new interaction partner of the NoxA complex in *B. cinerea*. Mutants that lack this ER chaperone display overlapping phenotypes to mutants of the NoxA signaling pathway. The protein appears to be involved in all major developmental processes, such as the formation of sclerotia, conidial anastomosis tubes and infection cushions (IC’s) and is needed for full virulence. Moreover, expression analyses and reporter gene studies indicate that BcPdi1 affects the redox homeostasis and unfolded protein response (UPR)-related genes. Besides the close association between BcPdi1 and BcNoxA, interaction studies provide evidence that the ER protein might likewise be involved in Ca^2+^ regulated processes. Finally, we were able to show that the potential key functions of the protein BcPdi1 might be affected by its phosphorylation state.

## Introduction

Plant pathogenic fungi threaten harvest yields of important economic crops in an increasing manner (Elad et al., 2016). One of the most devastating pathogens, which is responsible for tremendous crops losses of edible fruits and vegetables, is *Botrytis cinerea*, causative agent of the gray mold disease. The fungus has a necrotrophic lifestyle and benefits from high humidity and moderate temperatures. During vegetative growth, as well as during infection of plant material, various signaling molecules and cascades contribute to the developmental processes. Besides abiotic factors such as light and temperature (Tan and Epton, 1973; Broome et al., 1995; Schumacher et al., 2012), internal pathways determine the infection process. Central molecules within pathogenic processes are reactive oxygen species (ROS). ROS are present in all cells that depend on molecular oxygen: they are known to work as signaling molecules at appropriate concentrations, but harmful to macromolecules when present at high concentrations (Beckman and Ames, 1998).

Although plants produce ROS as the first defensive line against pathogen attack, *B. cinerea* produces its own ROS in highly conserved processes to induce the plant’s oxidative burst so that the fungus achieve full pathogenicity (Govrin and Levine, 2000; Heller and Tudzynski, 2011). While ROS result from the activity of the respiratory chain merely as by-products, there are enzymes, such as the NADPH oxidase (Nox) complexes, which actively form superoxide (O_2_^-^) by transporting electrons across the lipid bilayer on to molecular oxygen (Lambeth, 2004; Tudzynski et al., 2012). Nox complexes have been characterized most thoroughly in the mammalian system (reviewed in Laurindo et al., 2014). However, despite the evolutionary distance, there are several homologies between mammalian and fungal Nox complexes. For example, homologs in *B. cinerea* were identified for the catalytic subunit (gp91phox – BcNoxA/B), the regulatory subunit (p67phox – BcNoxR), adaptor proteins (p22phox – BcNoxD) as well as for putative transient members of Nox complexes such as the scaffold protein IQGAP (BcIqg1) (Segmueller et al., 2008; Siegmund et al., 2013, 2015; Marschall and Tudzynski, 2016a). Additionally, mammalian and fungal Nox complexes were shown to have functions in different cellular compartments with a changing complex composition dependent on the respective developmental process (reviewed in Laurindo et al., 2014; Marschall and Tudzynski, 2016b; Marschall et al., 2016a). While the mammalian Nox complexes are involved in cancer (Roy et al., 2015), Alzheimer’s disease (Wilkinson and Landreth, 2006) and atherosclerosis (Madamanchi and Runge, 2010), the Nox complexes of *B. cinerea* contribute to a wide range of vegetative and pathogenic processes, such as the formation of infection structures and sporulation (reviewed in Marschall and Tudzynski, 2016b). However, despite major advances made in recent years regarding the composition and function of fungal Nox complexes, there are still many questions about them that remain unanswered.

One of the central questions concerns the link between fungal Nox complexes and calcium (Ca^2+^) signaling processes. Since analyses in plants reveal a tight and recurrent connection between ROS and Ca^2+^ signaling pathways (Steinhorst and Kudla, 2014) many studies aim to unravel putative signaling hubs in fungi. Most recently, we were able to show that the protein BcIqg1 interacts with members of ROS and Ca^2+^ signaling cascades, though it remained unresolved whether the protein contributes to both pathways at the same time or independently (Marschall and Tudzynski, 2016a). Apart from the latter aspects, we have also unraveled the connection between both signaling pathways and the level of redox-mediated processes. Thus, we highlighted that a cytosolic oxidative burst is (1) dependent on both Nox catalytic subunits in *B. cinerea* and (2) that this oxidative burst appears to rely on elevated Ca^2+^ levels. Moreover, we elucidated that increasing Ca^2+^ concentrations promote a slight oxidative burst inside the endoplasmic reticulum (ER), mediated by the catalytic subunit BcNoxA (Marschall et al., 2016b). The ER is commonly accepted as a Ca^2+^ storage compartment (Koch, 1990), and previous studies established that both Nox complexes have distinct functions at the ER membrane (Siegmund et al., 2015; Marschall et al., 2016a). It is reasonable to suggest therefore that the ER plus its associated proteins may be a central location for the transmission of signals, and for the physical interaction of proteins belonging to ROS and Ca^2+^ signaling cascades.

The main function of the ER is the processing of (especially secretory) proteins by the oxidative protein folding machinery. Primarily responsible for the reduction, isomerization or oxidation of proteins within this process is the protein disulfide isomerase (PDI). In its oxidized form, PDI is able to function as a posttranslational modifying chaperone, which facilitates correct protein folding and catalyzes disulfide exchange reactions. During this process PDI is reduced and has to be regenerated by the oxidoreductin 1 (Ero1) (Kersteen and Raines, 2003). PDIs are highly conserved proteins that are involved in various developmental processes. Whereas in plants they are known to affect the development of endosperm (Li and Larkins, 1996) and gene expression in chloroplasts (Kim and Mayfield, 1997), in mammalian systems they were shown to influence intracellular Ca^2+^ concentrations (Corbett and Michalak, 2000; Lucero et al., 1998), apoptotic processes (Ko et al., 2002) and stress signaling (Sullivan et al., 2003). In mammals, however, the proteins are associated or directly interact with several important proteins of essential signaling pathways, such as calreticulin (Ca^2+^ signaling, Baksh et al., 1995) or members of the Nox complexes (Janiszewski et al., 2005; Laurindo et al., 2008; Fernandes et al., 2009; de A Paes et al., 2011).

In fungi, studies on PDIs showed that, due to its folding properties, the protein is involved in the secretory pathway (Ngiam et al., 2000), as well as being activated by exposure to stress (Saloheimo et al., 1999). Therefore, the protein clearly belongs to a large set of proteins, which are upregulated under ER stress and during the unfolded protein response (UPR) across all species boundaries (Haefliger et al., 2011; Perri et al., 2016). Moreover, the protein is essential for viability in *Saccharomyces cerevisiae* (Farquhar et al., 1991). However, information about interaction partners of PDI and its involvement in existing signaling networks is lacking.

Here, we report the occurrence of physical interactions between members of different signaling pathways. The close association of the PDI BcPdi1 to Ca^2+^ and ROS signaling cascades was determined by studying Δ*bcpdi1* mutants in comparison to deletion mutants of the Nox and Ca^2+^ signaling pathways. Many overlapping phenotypes in both vegetative and pathogenic processes were observed. The protein BcPdi1 appears to be essential for all major developmental processes, such as the formation of sclerotia, conidial anastomosis tubes (CATs) and infection cushions (IC’s), as well as for full virulence. Finally, we were able to show that the potential key function of the protein BcPdi1 may be affected by its phosphorylation state, which likewise is necessary for the maintenance of the redox homeostasis and the transmission of signals.

## Materials and Methods

### Cultivation of *Botrytis cinerea*

*Botrytis cinerea* Pers.:Fr. [*Botryotinia fuckeliana* (de Bary)Whetzel] B05.10 was collected from *Vitis vinifera* (Buettner et al., 1994) and was used in this study as basis strain and control in all experiments. Further strains are listed in **Supplementary Table S1**.

For cultivation synthetic complete medium (CM) (Pontecorvo et al., 1953) was used. For transformation, the strains were grown on PDAB medium (Potato dextrose agar [Sigma–Aldrich Chemie, Steinheim, Germany] supplemented with 100 g/l homogenized leaves of French beans (*Phaseolus vulgaris*). Cultures were grown for 6–8 days at 20°C under light conditions (12 h light/12 h darkness, full spectrum light) to obtain conidia. Sclerotia were induced by incubating the strains for 3 weeks at 20°C in darkness. For DNA preparation, the strains were grown for 3–4 days at 18°C on CM agar overlayed with Cellophane. For stress experiments, the CM was supplemented with agents inducing osmotic, oxidative, cell wall, membrane or ionic stress. Minimal medium was prepared after Czapek Dox (20 g/l sucrose, 3 g/l NaNO_3_, 1 g/l K_2_HPO_4_, 0.5 gl KCl, 0.01 g/l FeSO_4_ × 7 H_2_O, 0.5 g/l MgSO_4_ × 7 H_2_O, pH 5.2).

### Generation of Mutant Strains

For the generation of deletion and complementation constructs, the yeast homologous recombination system was used (Colot et al., 2006; Schumacher, 2012). For generating the deletion cassette of *bcpdi1*, the 5′/3′ flank were amplified with the primer 1/2 and 3/4 (**Supplementary Table S2**) as well as the resistance cassette with the primer pair 16/17. Recombinational cloning was done with *S. cerevisiae* FY834 and the vector backbone of pRS426 (*Eco*RI/*Xho*I). For complementation, *bcpdi1* was expressed under its native promotor (primer 10/15) as well as under the constitutive *oliC* promotor of *Aspergillus nidulans* (primer 9/10). The constructs for introducing mutated phosphorylation sites were amplified using the primer 9/12 and 10/11 (Δ*bcpdi1*-C-M1), the primer 9/14 and 10/13 (Δ*bcpdi1*-C-M2), 9/12, 11/14 and 10/13 (Δ*bcpdi1*-C-M1/2), 10/29 and 9/28 (Δ*bcpdi1*-C-DA). For the generation of PDI *in loco* complementation constructs that were fused to the genetically encoded biosensor roGFP2, six different fragments were generated by the primer pairs 1/11, 12/32, 33/34, 35/36, 37/38, 39/4 (PDI_phospho_null) or 1/28, 29/30, 31/32, 33/34, 35/36, 37/4 (PDI_phospho_mimic).

After transforming *S. cerevisiae* FY834, positive transformants were selected on SD medium lacking uracil since the strain is auxotrophic for this amino acid. Total DNA was isolated and transformed in *Escherichia coli*. Re-isolation was done by the Nucleo spin^®^ plasmid easypure kit (Macherey-Nagel, Düren, Germany) and correct assembly was tested by sequencing.

*Botrytis cinerea* was transformed with 40–60 μg of plasmid DNA as described previously (Gronover et al., 2001). Selection was done via hygromycin (70 μg/ml of hygromycin B (Invitrogen, San Diego, CA, United States) or nourseothricin (50 μg/ml of nourseothricin (Werner-Bioagents, Jena, Germany). Positive transformants were purified by single spore isolation: Conidia were spread on the appropriate selective medium; afterward, germinated conidia were picked and cultivated on new selective plates. Genomic DNA isolation was performed according to Cenis (Cenis, 1992). Correct and ectopic integrations were checked by diagnostic PCR and Southern blot (primer 18–27).

### Growth and Pathogenicity Assays

For characterization of the different mutant strains various growth, differentiation and pathogenicity assays were performed. For testing defects in pathogenicity, French bean plants (*Phaseolus vulgaris* L.) were infected according to Klimpel et al. (2002) with agar plugs or conidia from freshly sporulated PDAB plates. Germination on glass surfaces was tested as described by Doehlemann et al. (2006). Penetration ability was checked on onion epidermal layers after inoculation with washed conidia. Before microscopy, the hyphae growing on top of the layers were stained with lactophenol blue. For observation of ICs, the epidermal layers were inoculated with agar plugs of mycelium and incubated overnight (Siegmund et al., 2015). For visualization of CATs media and strains were prepared following Siegmund et al. (2015).

### Northern Blot Analysis

For Northern blot analyses, strains were grown on CM overlayed with Cellophane for at least 3 days. The mycelium was harvested, lyophilized and the RNA was isolated via the Trizol procedure (Invitrogen, Groningen, The Netherlands). Samples of 20 μg were loaded to an agarose gel and transferred to Hybond-N+ membranes. Northern blot hybridizations were done according to the method of Church and Gilbert (1984). The different probes used for expression analysis were generated by PCR with the primer pairs 40/41 (Hac1) and 42/43 (BiP/Kar2).

### Epifluorescence Microscopy

Light microscopy imaging was performed using the Axio Imager 2 and the Axiovert (Zeiss, Jena, Germany). Visualization of ICs was done with the 20× objective lens, while germinated conidia were analyzed with 40× or 63× magnification. ER staining was accomplished using the ER-Tracker^TM^ Blue-White DPX (Life Technologies, Germany) in McIlvaine standard buffer (McIlvaine, 1921). The samples were observed via the filter set 49 DAPI shift free (excitation G 365, beam splitter FT 395, emission BP 445/50). GFP fluorescence was detected with filter set 38 (excitation BP 470/40, beam splitter FT 495, emission BP 525/50). Images were captured with a Zeiss AxioCamMRm camera and further processed using the Axiovision Rel 4.8 software package.

### Confocal Laser Scanning Microscopy (CLSM) Imaging and Ratiometric Analysis

roGFP2 measurements were done using an inverted microscope (Leica DMIRE2) equipped with a Leica TCS SP2 scan head (Leica Microsystems, Wetzlar, Germany). Conidia were prepared as described previously (Heller et al., 2012). Results were obtained by using the excitation wavelengths 395 (first track) and 488 (second track) as well as a 505–530 bandpass filter for collecting images. Z-stacks were displayed as average projections via the CLSM software. Further evaluation was done with the Image J program (v.1.44f^1^) as it was shown before (Marschall et al., 2016b).

### Interaction Studies

Protein–Protein interactions were investigated by a split-ubiquitin based yeast-two-hybrid system (Dual System Biotech). The proteins of interest were fused to the C- or N-terminal half of ubiquitin. Upon interaction, ubiquitin congregates and the transcription factor controlling reporter genes (histidine and adenine – see also manual Dual Systems Biotech, Schlieren, Switzerland) can be released. Proteins without transmembrane regions are additionally fused to a transmembrane domain of the oligosaccharyltransferase subunit 4 (Ost4) of *S. cerevisiae* for anchoring the proteins at the membrane. Interaction vectors (prey/bait) were generated according to the manufacturer’s protocol. Both vectors (pPR3N = prey; pDHB1 = bait) were restricted with *Sfi*I and ligated with likewise restricted PCR products (for details see **Supplementary Table S2**). The ligated products were transformed in *E. coli* and selected with the appropriate selection marker.

For interaction assays, prey and bait were transformed in *S. cerevisiae* NMY51 according to the manufacturer’s protocol (DUALSystems Biotech) with a modified version of the lithium-acetate method (Gietz and Woods, 2002). Yeast cells were selected on SD-agar lacking the appropriate amino acids (*^-^leu^-^trp* as control of vector transformation; *^-^leu^-^trp^-^his^-^ade* as test for positive interaction). For drop tests, established colonies were grown overnight in SD medium lacking leucine and tryptophan. OD_600_ was adjusted to 1 and cells were pelleted. The medium was removed and starvation was induced by the addition of sorbitol (1 M): incubation then took place for at least 5 h at 30°C and 200 rpm on a shaker. Subsequently samples were diluted up to 1:1000 and 10 μl of each dilution were dropped on selective medium (SD*^-^leu^-^trp^-^his^-^ade* + 5 mM 3-AT + 80 mg/l X-Gal) and control plates (SD*^-^leu^-^trp*). Plates were incubated for 3–4 days at 30°C.

For co-IP experiments, proteins of interest were transformed into the wild type strain. The expression is controlled by the constitutive *oliC* promotor (*A. nidulans*) and reporter genes were fused to the N- or C terminus of the respective protein. Fusion proteins were generated by the yeast recombination system. Whereas *bcnoxA* was cloned into the vector pNAH_OCT (restricted with *Nco*I) containing *mcherry* as reporter gene, *bcpdi1* was cloned into the vector pNDN_OGG (restricted with *Nco*I) with *gfp* as fluorescent marker.

After transformation, the strain expressing both constructs was purified by single spore isolation and grown on CM medium with Cellophane overlay for 3–4 days. The mycelium was harvested, lyophilized and proteins were isolated by grinding and applying disruption buffer [20 mM Tris/HCL pH 8, 150 mM NaCl, 0.05% Triton X-100 supplemented with 10 μl Phosphatase inhibitor 1/2 and 10 μl of Protease inhibitor (Sigma–Aldrich, Steinheim, Germany)]. For the NoxA_mcherry control, the extract was stored until Western blotting. The protein extract of the PDI_GFP control as well as of the strain expressing both constructs was loaded to μMACS GFP beads/columns and purified according to the instruction manual of the μMACS GFP tagged protein isolation kit (Milteny Biotech Bergisch Gladbach, Germany). After Western blotting, detection was accomplished using the anti-gfp antibody (Milteny Biotech, Bergisch Gladbach, Germany). For mcherry detection, the anti-mcherry antibody (Thermo Fisher Scientific, Schwerte, Germany) was used as primary antibody and for the secondary antibody the donkey-anti-rabbit antibody (Thermo Fisher Scientific, Schwerte, Germany) was used. Visualization was conducted using the enhanced chemi-luminescence (ECL) detection (Bio-Rad, Munich, Germany).

### Database Resources

Nucleotide and protein sequences of *B. cinerea* strain B05.10 were obtained by the database Ensembl^2^. For the analysis of the sequences, different programs were used: Signal peptides were predicted by SOSUIsignal (Gomi et al., 2000), subcellular localization patterns of proteins were predicted by ProtComp v.9.0^3^, transmembrane regions were predicted by using the programs TMHMM^4^ and SACS MEMSAT2 (Jones et al., 1994). Phosphorylation sites were predicted by KinasePhos (Huang et al., 2005).

## Results

### Functional Analysis of the Protein Disulfide Isomerase BcPdi1

Since our studies had revealed that the NoxA complex has a distinct function inside the ER, putative targets/partners were investigated. An essential ER protein, which in mammalian systems is associated with subunits of the Nox complex (Janiszewski et al., 2005; Laurindo et al., 2008; Fernandes et al., 2009; de A Paes et al., 2011) is the PDI. Furthermore, the putative homolog to the mammalian protein was identified as a putative interaction partner of BcNoxA in a cDNA library screen (Siegmund and Tudzynski, unpublished). Blastp analysis and multiple sequence alignments using ClustalΩ (Sievers et al., 2011) displayed a query coverage of 85% as well as a sequence identity of 37% by comparing the mammalian PDI with the fungal protein (Bcin06g05730). According to the Ensemble *Botrytis* genome database^5^ the ORF consists of 1596 bp encoding a protein of 531 aa. Bioinformatic analyses revealed that the protein (BcPdi1) probably contains two transmembrane domains with a small cytosolic loop (**Supplementary Figure S1A**), with both ends located inside the ER. Its two catalytic domains (CXXC-motif) are predicted to be located in the cytosolic loop (1 in **Supplementary Figure S1A**) and inside the ER, respectively (4 in **Supplementary Figure S1A**). Moreover, two cytosolic phosphorylation sites are predicted (2/3 in **Supplementary Figure S1A**). Interestingly, the first putative phosphorylation site is highly conserved among different fungal species (**Supplementary Figure S1A**) while the second one is poorly conserved.

For functional characterization, deletion mutants of *bcpdi1* were generated (**Supplementary Figure S1B**). Transformants were checked via diagnostic PCR (**Supplementary Figure S1B**) and Southern blot analysis (**Supplementary Figure S1C** – for details see **Supplementary Tables S1**, **S2**). Since all mutants displayed an identical phenotype, the results of the mutant Δ*bcpdi1*_T2 are presented. In order to elucidate the role of the predicted cytosolic phosphorylation sites of the protein, mutants with mutated phosphorylation sites were generated. The key amino acids were replaced by an alanine to mimic the constitutively dephosphorylated state (phospho null) (BcPdi1_M1 = T65A; BcPdi1_M2 = Y208A). Moreover, the putative first phosphorylation site was transferred into its constitutively phosphorylated form (phospho mimic) by replacing threonine by glutamic acid (T65E).

### BcPdi1 Is Involved in Oxidative and Osmotic Stress Resistance

To check whether BcPdi1 is involved in stress response pathways, the radial growth was determined on CM containing various stress agents.

The radial growth of the Δ*bcpdi1* mutant on CM was reduced by 20% in comparison to the wild type. Whereas the complemented strain, Δ*bcpdi1*-C-M2 and Δ*bcpdi1*-C-DA grew as well as the wild type, the colony diameters of Δ*bcpdi1*-C-M1 (87.5%) and Δ*bcpdi1*-C-M1/2 (83.5%) were slightly reduced.

**FIGURE 1:**
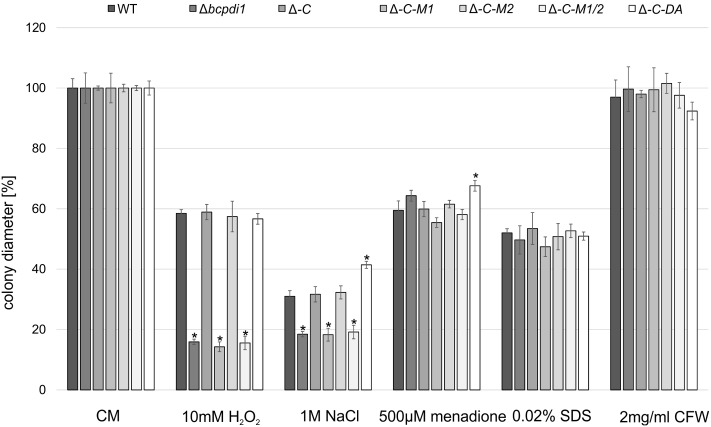
BcPdi1 mediates stress resistance against oxidative and osmotic stress. Agar plugs of 3-days-old CM plates were incubated on CM plates supplemented with 10 mM H_2_O_2_, 1 M NaCl, 500 μM menadione, 0.02% SDS or 2 mg/ml Calcofluor White. Colony diameters were measured and compared to the wild type (here after 3 days). Replicates showed similar results. Mean values and standard deviations were calculated from three independent experiments. Asterisks indicate significant differences to the wild-type control in every condition (*t*-test, *P* < 0.01).

The deletion of *bcpdi1* led to a higher sensitivity of the mutants to oxidative and osmotic stress. The addition of 10 mM H_2_O_2_ and on 1 M NaCl was sufficient to reduce the rate of radial growth of Δ*bcpdi1* significantly, but the addition of menadione had no effect. While the complemented strain displayed wild type-like growth on all selective media, the mutation of the first predicted phosphorylation site correlated with the deletion mutant’s phenotype. The mutation of the second putative phosphorylation site had no effect (**Figure 1**). The ‘phospho mimic’ mutants mostly resembled the growth behavior of the wild type. However, on NaCl-supplemented medium the mutant grew much better than all other strains. In contrast to oxidative and osmotic stress, the addition of Calcofluor White (2 mg/ml) as a cell wall stressor, or SDS (0.02%) as a membrane stressor did not differentially influence the radial growth of the mutants compared to the wild type.

### BcPdi1 Affects Production of Conidiospores and Virulence

*Botrytis cinerea* is able to infect its host from spore- or mycelium-mediated penetration processes. Whereas appressoria are formed after the short germination of a fungal spore, so-called ICs are mycelium-derived penetration structures that facilitate the breakthrough of the plant cell wall (Armentrout and Downer, 1987).

**FIGURE 2:**
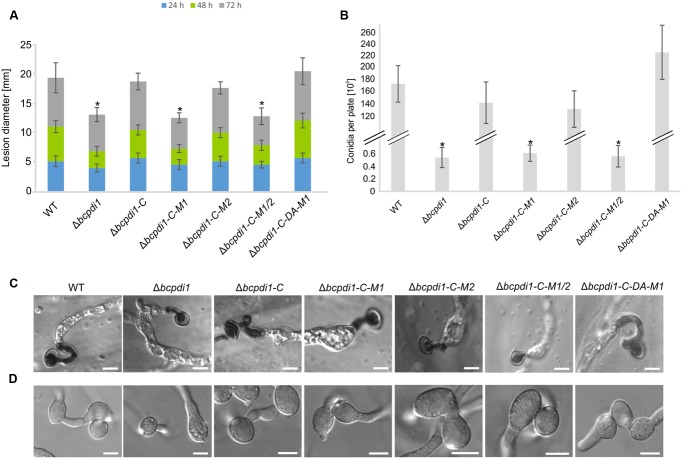
The deletion of *bcpdi1* impacts spore mediated infection, CAT and conidia formation. **(A)** Primary bean leaves were inoculated with conidia (10^5^). Lesion diameters were measured each day and evaluated statistically. Significant differences were indicated by asterisks (*t*-test, *P* < 0.01). Replicates showed similar results. **(B)** Mutant strains of *bcpdi1* are impaired in the production of conidiospores. Agar plugs were incubated on CM plates for at least 3 weeks. Conidia were washed down and counted. Results of five biological replicates were similar. Mean values and standard deviations were calculated from five independent experiments. Significant differences to the wild type control were indicated by asterisks (*t*-test, *P* < 0.01). **(C)** The deletion of *bcpdi1* leads to defects in hyphal fusions. Conidia were plated on Vogel’s minimal medium and incubated for 18 h. For each strain 300 conidia were investigated concerning their fusion ability. **(D)** The penetration ability is unaltered in the mutants of *bcpdi1*. Onion epidermal layers were inoculated with conidia. Just before microscopy, lactophenol blue staining was performed to visualize the fungal material on the surface of the onion layers.

The deletion of *bcpdi1* attenuated the spore-mediated infection of bean leaves, but the way the mutant formed appressoria appeared as in the wild type (**Figures 2A,C**). For this study, spores were inoculated on onion epidermal layers and fungal material was stained with lactophenol blue after 16 h, allowing discrimination between the stained surface hyphae and those left unstained after penetration of the host. The mutants were able to penetrate the plant’s surface but the subsequent colonization was severely inhibited (**Figure 2A**). A similar phenotype was recorded for the strains Δ*bcpdi1*-C-M1 as well as Δ*bcpdi1*-C-M1/2. In marked contrast, the complemented strain (Δ*bcpdi1*-C) and Δ*bcpdi1*-C-M2 appeared to infect in the manner of the wild type.

All strains that displayed a retarded colonization of plant tissue produced significantly fewer spores than the wild type and the strains that restored the wild type phenotype (**Figure 2B**). However, since all plants were infected with the same concentration of conidiospores, there is no causal correlation between reduced production of conidia and impaired virulence.

**FIGURE 3:**
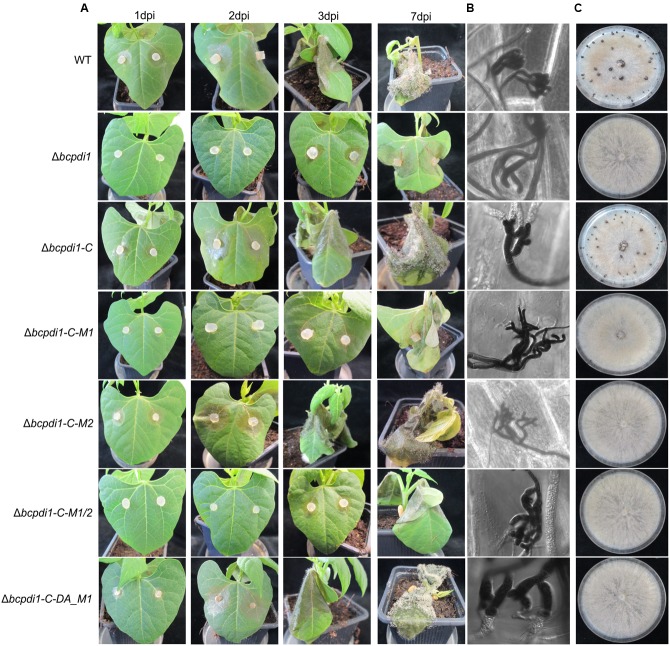
The deletion of *bcpdi1* influences mycelium mediated infection and the production of sclerotia. **(A)** Primary bean leaves were incubated with agar plugs from 3-day-old CM plates. Lesion diameters were measured and analyzed statistically. Biological replicates behaved similar. **(B)** The production of infection cushions was altered in mutants of *bcpdi1*. Agar plugs were incubated on onion epidermal layers for at least 20 h. Lactophenol blue staining was accomplished to detect fungal material that is located on the surface of the onion layers. **(C)** BcPdi1 affects the production of sclerotia. CM Plates with agar plugs were incubated in constant darkness for at least 14 days. Biological replicates behaved similarly.

When studying the mycelium-mediated infection process of *B. cinerea* similar results were obtained as previously for the spore-mediated infection. In contrast to the wild type and the strains Δ*bcpdi1*-C and Δ*bcpdi1*-C-M2, the deletion mutant was impaired in its ability to achieve mycelium-mediated penetration as well as in the colonization stage (**Figure 3A**). Primary lesions were not visible until the 3rd day of infection. Similar results were obtained for Δ*bcpdi1*-C-M1 and Δ*bcpdi1*-C-M1/2.

The defects in infecting plant tissue may be due to the impaired production of ICs. All strains that were unaffected in the mycelium-mediated infection process were still able to produce ICs. In contrast, the mutant strains Δ*bcpdi1*, Δ*bcpdi1*-C-M1, and Δ*bcpdi1*-C-M1/2 were unable to produce wild type-like ICs, but instead produced twisted hyphae which were not targeted toward one infection site as is normal for the wild type (**Figure 3B**).

### BcPdi1 Influences the Formation of Hyphal Fusions and Sclerotia

Since it has been shown previously that members of the NoxA complex as well as putative interaction partners are impaired in the formation of CATs (Roca et al., 2012; Siegmund et al., 2015; Marschall and Tudzynski, 2016b), the different PDI strains were investigated with respect to this differentiation process. These specialized hyphae are formed to exchange cellular material between two hyphae of the same organism. Whereas the wild type, as well as the strain Δ*bcpdi1*-C, were still capable of forming CATs, the deletion mutant and the strains Δ*bcpdi1*-C-M1, Δ*bcpdi1*-C-M1/2, and Δ*bcpdi1*-C-DA were impaired in the formation of hyphal fusions. The mutant Δ*bcpdi1*-C-M2 was still able to form the hyphal structures, but produced markedly less CATs than the wild type (**Figure 2D**).

Apart from hyphal fusions, BcPdi1 also affects the production of perennial survival structures called sclerotia. These structures, which are produced under constant darkness, were formed by the wild type and the complemented strain Δ*bcpdi1*-C (**Figure 3C**). All other strains were defective in the production of sclerotia. Surprisingly, the mutant strain Δ*bcpdi1*-C-M2 also failed to complement the defect in sclerotial development, although this mutant behave as the wild type in all other respects.

To summarize, BcPdi1 affects important vegetative and pathogenic processes, such as sclerotial formation, hyphal fusions and penetration structures. Essential for these developmental processes is the cytosolic region of BcPdi1 containing the two putative phosphorylation sites as well as one catalytically active site. Whereas the second predicted phosphorylation site seems to have an exclusive effect on the production of sclerotia, the first site seems to be essential for all essential protein functions.

### BcPdi1 Localizes to the ER

To study the localization pattern of BcPdi1, a GFP fusion construct was generated under the control of the native promoter (1 kb upstream of the ORF) and the constitutive *oliC* promoter. Both were transformed into the deletion mutant’s background. The wild type phenotype was fully restored by both complementation constructs (Data not shown). Additionally, in both cases the target protein localized to identical structures. The strain BcPdi1-C was used since the fluorescence of the protein under the constitutive *oliC* promoter was much brighter for localization studies and later experiments. Apart from re-integrating *bcpdi1* into its wild type version, GFP-fusion constructs with the mutated alleles (Δ*bcpdi1*-C-M1, Δ*bcpdi1*-C-M2, Δ*bcpdi1*-C-M1/2, Δ*bcpdi1*-C-DA-M1) were generated.

**FIGURE 4:**
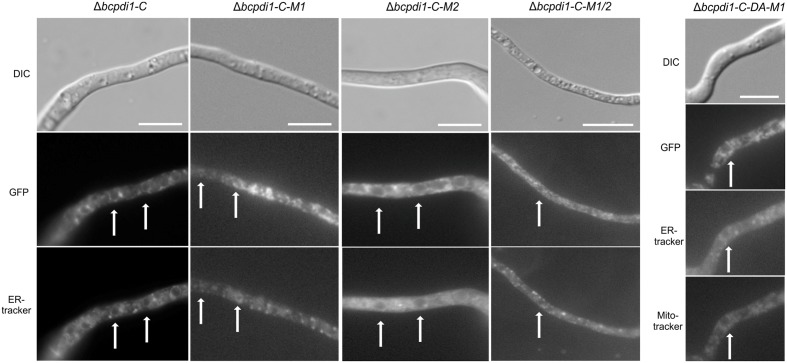
BcPdi1 localizes to ER structures. Conidia of the strains expressing fusion constructs of BcPdi1 and GFP were cultivated overnight on microscopic slides in B5 medium supplemented with glucose (2%). GFP fluorescence is visible in filamentous ER structures and inside the nuclear envelope. Dependent on the inserted mutation inside the BcPdi1_GFP fusion construct (mimicking [de]phosphorylated) the fluorescence is shifted from the perinuclear localization to filamentous structures, which partially overlap with mitochondrial structures. Co-staining was performed with Hoechst 33342 and trackers for the detection of the ER (Blue-White DPX) or mitochondria (mitoIDred). DIC, differential interference contrast microscopy.

BcPdi1 localized to filamentous structures (**Figure 4**) belonging to the ER (verified by co-staining with ER tracker) and was additionally found in the nuclear envelope (verified by co-staining with the DNA dye Hoechst 33342 – Data not shown). Although the mutation of the second putative phosphorylation site (M2) into its phospho null form had no influence on the localization, the pattern changed upon mutation of M1. BcPdi1_M1 was detected in a faint network of filamentous structures belonging to the ER, but was hardly visible in the nuclear envelope. Moreover, the proteins seemed to adhere as aggregates that were detected throughout the hyphae. Similar results were obtained for the BcPdi1_GFP fusion construct containing both mutated phosphorylation sites.

The localization pattern of the phospho mimic mutants differed from all other observed PDI-constructs. The fusion constructs localized to filamentous structures, which show high similarity to mitochondria. Co-localization with the mitoIDred mito tracker as well as with the ER tracker revealed that those structures are overlapping with ER and mitochondrial structures (**Figure 4**).

In summary, we have established that BcPdi1 is indeed an ER protein that is located within the nuclear envelope and filamentous structures. Mutations of the first predicted cytosolic phosphorylation site of the protein directly influenced the distribution of the protein within hyphae, a most unexpected result.

### BcPdi1 Affects the Cytoplasmic Redox State

**FIGURE 5:**
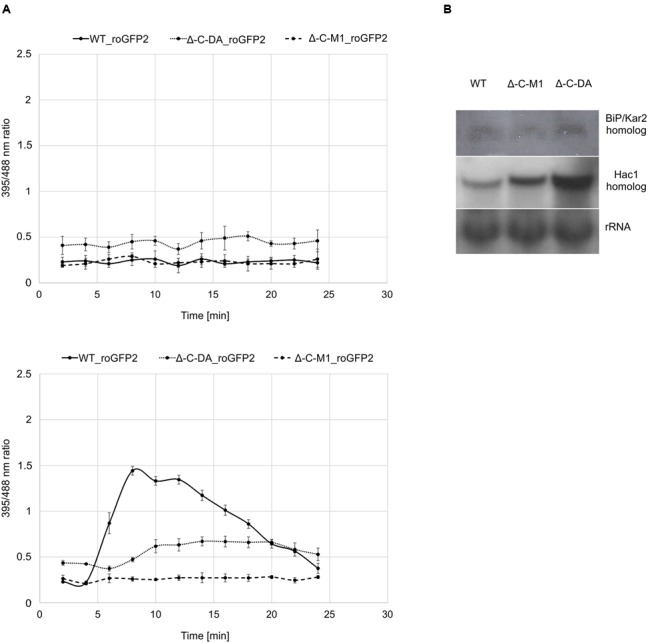
Protein disulfide isomerase has an impact on the cytosolic redox state and the UPR. **(A)** Cytosolic roGFP2 constructs were transformed and expressed into Δ-C-M1 and Δ-C-DA. Z-stacks of images were taken by CLSM with excitation at 395 and 488 nm. Ratio images were generated using the ImageJ software. The _395/488_ratio inside the cytosol of the mutants was compared to the wild type under non-inducing conditions (above) and upon induction with 50 mM CaCl_2_ (below) after 4 min. Mean values and standard deviations were calculated from five biological replicates. **(B)** Expression analysis of BiP/Kar2 and Hac1 homologs in the wild type and the mutants Δ*bcpdi1*-C-M1_roGFP2/Δ*bcpdi1*-C-DA_roGFP2 via Northern blot studies. Strains were grown on CM medium overlayed with Cellophane for 3 days. RNA was isolated and expression was determined by Northern blotting. The rRNA probe was used as loading control.

To elucidate the role of BcPdi1 both inside and outside the ER, studies with the genetically encoded biosensor roGFP2 were performed. The modified version of the reporter gene was suitable for the visualization of the glutathione pool, which mirrors the current redox state of the monitored compartment (Schwarzlaender et al., 2008; Heller et al., 2012). These studies aimed to establish, whether BcPdi1 affects the cytosolic redox state and, therefore, is involved in the maintenance of the intracellular redox balance. Under non-inducing conditions, the phospho null version (Δ*bcpdi1*-C-M1_roGFP2) appeared like the wild type and the deletion mutant (data not shown) whereas the phospho mimic version (Δ*bcpdi1*-C-DA_roGFP2) exhibited an elevated _395/488_ratio (**Figure 5A** top).

Since previous studies with mutants of both Nox complexes revealed that CaCl_2_ is sufficient to induce an oxidative burst inside the cytosol, the mutants (Δ*bcpdi1*-C-M1_roGFP2; Δ*bcpdi1*-C-DA_roGFP2) were also tested under those conditions (50 mM CaCl_2_). Surprisingly, both patterns of the mutants did not resemble the wild type control. In the wild type, the addition of CaCl_2_ induced a massive oxidative burst, changing the _395/488_ratio from 0.3 to 1.4. In comparison, the _395/488_ratio of Δ*bcpdi1*-C-M1_roGFP2 remained unaffected as the deletion mutant Δ*bcpdi1* (data not included in the figure, identical curve), and the _395/488_ratio of the phospho mimic version (Δ*bcpdi1*-C-DA_roGFP2) was slightly shifted to a more oxidized state than the wild type (**Figure 5A** below). The induction via CaCl_2_ led only to minor changes of the _395/488_ratio.

To elucidate, why the basic _395/488_ratio of Δ*bcpdi1*-C-DA_roGFP2 increased in comparison to the wild type, we did expression studies with known marker genes of the UPR. Since previous studies in plant cells revealed that changes in the redox state are due to the activity of the UPR machinery (Merksamer et al., 2008) we analyzed the *bcpdi*1 mutants expressing roGFP2 with respect to UPR activity. We used the putative *B. cinerea* homologs of the genes BiP/Kar2 (Bcin13g00960) and Hac1 (Bcin08g06730). Whereas BiP/Kar2 is a chaperone responsible for the correct folding of proteins (Jung et al., 2013), the transcriptional activator Hac1 binds to UPR-responsive elements to increase the amount of ER-resident proteins required for the folding machinery as well as components of the secretory pathway (Mori et al., 1992, 1998). Both were analyzed in the wild type in comparison to Δ*bcpdi1*-C-M1_roGFP2 and Δ*bcpdi1*-C-DA_roGFP2. The expression of the BiP/Kar2 homolog appeared to be wild type like in both mutants. The Hac1 homolog was upregulated in Δ*bcpdi1*-C-DA_roGFP2 (**Figure 5B**). In summary, the results suggest that BcPdi1 is involved in the maintenance of the cytosolic redox state and may play a role in the UPR.

### BcPdi1 Interacts with Proteins of Nox-, Ca^2+^-, and Redox Signaling Pathways

**FIGURE 6:**
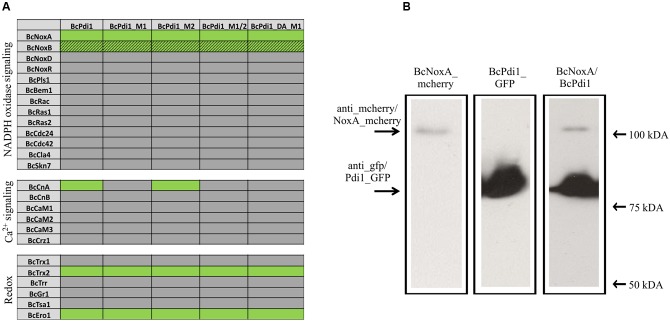
PDI interacts with proteins of different signaling pathways. **(A)** In yeast-two hybrid assays, the interaction of BcPdi1 with proteins of different signaling cascades was analyzed. Besides the unmutated version, isoforms were included with mimicked dephosphorylated phosphorylation sites (M1/M2/M1_2) or the mimicked phosphorylated site (PDI_DA_M1). All constructs with the respective bait/prey protein were transformed in *S. cerevisiae* NMY51 and plated/dropped on selective media (SD^-^leu^-^his^-^trp^-^ade + Xgal + 5 mM 3AT). If proteins interact, a growth on selective media will be possible. Positive interactions are illustrated in green, whereas lacing interactions were highlighted in gray. Streaky fields (gray/green coloration) indicate weak interactions. As negative controls, the respective empty prey/bait vector was co-transformed with the protein of interest. **(B)** BcNoxA and BcPdi1 interact in co-IP assays. For co-IP assays the strains expressing either BcNoxA_mcherry or BcPdi1_GFP were used as control strains. Interactions were analyzed with the strain B05.10 expressing both fusion constructs. After preparing the total protein extract, purification was achieved via μMACS GFP beads/columns. All extracts were separated by Western blotting (from left to right: B05.10 + NoxA_mcherry; B05.10 + BcPdi1_GFP; B05.10 + BcNoxA_mcherry and BcPdi1_GFP). Anti-GFP or anti-mcherry antibodies were used for detection. In Lane 3 both fusion proteins were labeled with both antibodies simultaneously (BcPdi1_GFP = 84 kDa; BcNoxA_mcherry = 102 kDa).

To examine whether the overlapping functions between the Nox complex and PDI are based on physical/direct interaction of these proteins we used a yeast-two hybrid approach; since membrane associated proteins are involved, we chose the split-ubiquitin system which is optimized for monitoring interactions between transmembrane proteins. The protein BcPdi1 was shown to interact with the catalytic subunit of the NoxA complex, and to a minor extent with BcNoxB (**Figure 6A** and **Supplementary Figure S2**). The positive interaction of BcNoxA and BcPdi1 was confirmed in co-IP experiments (**Figure 6B**). Strains expressing BcNoxA.mcherry (lane 1) or BcPdi1_GFP (lane 2) were used as controls (BcPdi1_GFP = 84 kDa; BcNoxA_mcherry = 102 kDa) (see also Materials and Methods). Additional interactions were shown for BcPdi1 with thioredoxin 2 (BcTrx2), an oxidoreductin (BcEro1) and the catalytic subunit of the calcineurin complex (BcCnA).

To elucidate the impact of the putative phosphorylation sites on the interaction with the different proteins, and also for the yeast-two hybrid based method, the putative cytosolic phosphorylation sites were mutated (BcPdi1_M1 = T65A; BcPdi1_M2 = Y208A). Again, the first putative phosphorylation site was transferred into its putatively dominant active form (phospho mimic) (T65E).

Interaction tests were done comparatively with the mutated versions of BcPdi1. While the mutation of the second predicted phosphorylation site (M2) had no effect on any of the interactions, mutation of M1 (T65A) and the phospho mimic led to the loss of the interaction with BcCnA. There was no effect on the binding to NoxA/B, Trx2, and Ero1. (**Figure 6** and **Supplementary Figure S2**).

To summarize these results it is clear that BcPdi1 interacts with members of various signaling cascades such as Nox-, Ca^2+^-, and redox related pathways. The catalytic subunit of calcineurin (BcCnA) seems to bind to the cytosolic region of the protein and in particular to a segment exhibiting a single putative phosphorylation site.

In conclusion, we present evidence that BcPdi1 is involved in all major developmental processes, such as the formation of conidia, sclerotia and CATs. Moreover, the ER protein affects the virulence of the necrotrophic plant pathogen and mediates stress resistance to oxidative and osmotic stress. The phenotype of Δ*bcpdi1* is similar to those of Δ*bcnoxA* and Δ*bcnoxD* mutants suggesting a link between Nox signaling and BcPdi1 in these differentiation processes. A cytosolic phosphorylation site, which is highly conserved among different species, clearly is essential for the function of BcPdi1. In its phospho null form, the respective mutants behave like Δ*bcpdi1*. In contrast, the phospho mimic mostly restores the wild type phenotype.

## Discussion

Reactive molecules derived from oxygen and the redox homeostasis are essential parameters contributing to the complex lifestyle of the plant pathogen *B. cinerea* (Heller et al., 2012; Marschall and Tudzynski, 2016b; Marschall et al., 2016b). Especially during the interaction with its host, the fungus has to cope with external stimuli and needs to adapt itself to the changing environment. Major signals during the plant-pathogen interaction are ROS; these chemical species are produced by plants as a defense reaction, and also generated by the fungus during the intimate interaction with its host. In fungi, one of the main sources of the reactive molecules are NADPH oxidase (Nox) complexes that are transporting electrons across the lipid bilayer on to molecular oxygen (Bedard and Krause, 2007; Tudzynski et al., 2012). Although there has been tangible progress in the elucidation of the fungal Nox complexes in recent years, there are still many open questions that arise concerning Nox functionality, composition and the integration into existing signaling cascades.

Recent studies in *B. cinerea* revealed that Nox complexes have distinct functions within the ER (Siegmund et al., 2015; Marschall et al., 2016a). The ER is an important cellular organelle since essential processes such as protein processing (translation, folding, and transport), the UPR and the storage of metabolites take place in here. In fungi, information is scarce about the ER-proteins that are mediating these processes.

In this study, we identified the ER protein BcPdi1 as new interaction partner of BcNoxA. In yeast-two hybrid experiments, as well as by co-immunoprecipitation, the ER protein was found to directly bind to the catalytic subunit of the NoxA complex. In other fungi, PDI is essential for viability (Farquhar et al., 1991) and is activated during ER-stress exposure, as in *Trichoderma reesei* (Saloheimo et al., 1999). Moreover, PDI is involved in the oxidative protein-folding machinery, which facilitates the formation of disulfide bonds (especially of secretory proteins) and the refolding of misfolded polypeptides. A direct interaction of BcPdi1 and BcNoxA might occur during the above-mentioned folding process dependent on the ER redox state and putative co-factors (Mezghrani et al., 2001). A more likely hypothesis is that the interaction of both proteins is dependent on certain differentiation processes, as described in mammalian cultures in which Nox activity was shown to be dependent on PDI (Laurindo et al., 2008, reviewed in Zeeshan et al., 2016). In *B. cinerea*, putative interaction sites are the ER-located loops of the transmembrane protein BcNoxA, but it remains unclear in which direction the interaction between both proteins takes place in fungi. It is possible that BcNoxA works downstream of BcPdi1 since the phenotype of Δ*bcpdi1* appeared to be more severely affected than the one of Δ*bcnoxA*. Therefore, the binding of BcPdi1 could regulate BcNoxA activity in an manner still unknown, and perhaps similar to the mammalian system (Laurindo et al., 2008). In contrast, BcNoxA might work upstream of BcPdi1 and could be responsible for the transfer of electrons from NADPH to BcPdi1. As a third possibility, the NoxA complex may actively produce superoxide, which is detoxified immediately by BcPdi1. Generation of superoxide would have the consequence that BcPdi1 would be shifted to its oxidized form, increasing its activity and thus allowing it to process a higher level of secretory proteins. This hypothesis is supported by findings that the levels of secreted proteins were altered in deletion mutants of *bcnoxA* and *bcpdi1* (data not shown).

Nevertheless, the obtained data support the hypothesis that both BcNoxA and BcPdi1 are acting in the same signaling pathway(s). Consequently, all major developmental processes are affected in a similar way. Besides the impact on ICs, conidiospore production and formation of CATs, both proteins impact on virulence and formation of sclerotia. The more severe impact on virulence of Δ*bcpdi1* may be attributed to the predicted two catalytic active domains located inside and outside of the ER. Therefore, the protein may influence signaling components which are located inside the ER, such as BcTrx2 (Viefhues et al., 2014), at the ER membrane, such as BcNoxA (Segmueller et al., 2008), as well as cytosolic components, such as BcCnA (**Figure 6**) (Harren et al., 2012). Since deletion mutants of *bctrx2* appeared to be wild type like (Viefhues et al., 2014), the observed defects in vegetative and pathogenic processes are not attributable to this interaction. In contrast, the close association of BcPdi1 with the catalytic subunit of the phosphatase BcCNA might be more important. In former studies in our laboratory, mutants lacking *bccna* were generated. They displayed severe growth defects and appeared to be avirulent (Harren et al., 2012). To unravel which protein works upstream/downstream of each other and to elucidate the binding mechanism between BcPdi1 and BcCnA, the PDI was analyzed bioinformatically. Surprisingly, only two phosphorylation sites were predicted in the cytosolic loop of BcPdi1. These sites may be putative target sites of regulating kinases and phosphatases. For both sites, mutants were generated mimicking the (de)phosphorylated version of the phosphorylation sites. Characterization revealed that the second predicted phosphorylation site (P-site) has no impact on binding to any interaction partner and had hardly any effect on growth and pathogenicity of the fungus (**Figures 1**–**3**). In contrast, the first putative P-site appeared to be essential for binding to the phosphatase and for every observed phenotype that had previously been described for the deletion mutant. It is noteworthy that both approaches, mimicking the phosphorylated and dephosphorylated version of the predicted P-site, are unable to restore growth typical of the wild type. Whereas the phospho null version resembles the phenotype of the deletion mutant, the phospho mimic version restored the wild type phenotype only partially. Thus, the results indicate that phosphorylation of BcPdi1 at the first predicted P-site is essential for its function, but it is remarkable that mimicking the phosphorylation does not have the opposite effect. The mutants displayed an intermediate phenotype between the deletion mutant (lack of CATs, sclerotia) and the wild type (stress sensitivity, virulence, and conidia production). This intermediate effect may be because the phosphorylation is only mimicked by the replacement of threonine by glutamic acid. Two indications that there may be even more complexity behind the phenotype was the localization of the protein and the cytosolic redox state of the PDI mutants. The protein localization of the BcPdi1_GFP fusion construct was monitored in the nuclear envelope as well as in filamentous structures of the ER (**Figure 4**). Whereas the mutation of the second putative P-site had no influence on the localization pattern, in the mutants mimicking the phosphorylation of the first predicted P-site the protein was localized in small filamentous structures and hardly detectable in the nuclear envelope. Co-staining with ER- and mito-tracker agents revealed that both compartments seem to overlap. This phenomenon has previously been observed in mammalian cells in which the formation of tight mitochondria-ER complexes was dependent on stress conditions and involved in the regulation of the redox homeostasis and cell death (Kornmann and Walter, 2010; Grimm, 2012; Marchi et al., 2014; Stoica et al., 2014). To verify the presence of similar processes in *B. cinerea*, we analyzed the cytosolic redox state in the wild type in comparison to all mutants. While the unstimulated redox levels in Δ*bcpdi1* (data not shown) and Δ*bcpdi1*-C-M1 appeared to be as in the wild type, the mutants mimicking the phosphorylation of the first predicted P-site exhibited a higher _395/488_ratio inside the cytosol. This unbalanced redox equilibrium became even more obvious under Ca^2+^ inducing conditions. Here in the wild type an oxidative burst is detectable upon Ca^2+^ stimulation. In previous studies, we were able to show that both Nox complexes contribute to the changes in redox status upon Ca^2+^ exposure. Surprisingly, the deletion mutant and Δ*bcpdi1*-C-M1 also seem to be insensitive to the addition of CaCl_2_, suggesting that BcPdi1 is part of the linear signal messaging of Ca^2+^ stress in close association to BcNoxA and BcNoxB. All mutants mimicking the phosphorylation of the first putative P-site displayed a very different pattern. A much higher _395/488_ratio and the absence of a wild type-like oxidative burst were monitored. To prove which cellular processes might contribute to this elevated _395/488_ratio, the expression of key genes of the UPR were analyzed (Graf et al., 2008; Jung et al., 2013; Heimel et al., 2013; Montenegro-Montero et al., 2015). Northern blot studies confirmed that the expression of the homolog to Hac1 was significantly upregulated in the mutants mimicking the phosphorylation of the first predicted P-site (**Figure 5**). Therefore, the mutation of the first putative P-site may hinder the function of the PDI so that the redox homeostasis is unbalanced and rescue programs, such as the UPR, might be induced.

In summary, we show in this work that BcPdi1 is an ER protein, which is involved in several growth and differentiation processes such as formation of CATs, ICs, conidia, sclerotia as well as in virulence of *B. cinerea*. The phenotype of the deletion mutant resembles the ones of Δ*bcnoxA* and Δ*bcnoxD* and display – together with the physical interaction of BcNoxA and BcPdi1 – a strong link between ROS signaling and ER proteins and processes. The evidence at present is inconclusive as to which direction certain signals were forwarded in the pathways. Starting points for further studies might be the different tie-points between ROS, the ER and Ca^2+^. Therefore, not only does the physical interaction of BcPdi1 and BcCnA hint to a rather close association, but even more to the collaboration of BcNoxA, BcNoxB and BcPdi1 upon exposure to Ca^2+^. Future studies should investigate how Ca^2+^ affects the regulation of Nox complexes, ER proteins and the redox homeostasis.

## Author Contributions

RM: conception, methodology, writing of the manuscript and realization of all experiments. PT: conception, review and editing. Final approval: RM and PT.

## Conflict of Interest Statement

The authors declare that the research was conducted in the absence of any commercial or financial relationships that could be construed as a potential conflict of interest.

## Footnotes

1 http://rsb.info.nih.gov/ij/

2 http://fungi.ensembl.org/Botrytis_cinerea/Info/Index

3 http://www.softberry.com/berry.phtml?topic=protcompan&group=programs&subgroup=proloc

4 http://www.cbs.dtu.dk/services/TMHMM/

5 http://fungi.ensembl.org/Botrytis_cinerea/Info/Index

## Supplementary Material

**FIGURE S1 |** Bioinformatics and *bcpdi1* deletion strategy. **(A)** Multiple sequence alignment of BcPdi1 homologs of “*Botrytis cinerea*,” “*Aspergillus niger*,” “*Neurospora crassa*,” and “*Sclerotinia sclerotiorum.*” Alignment was performed by using MegAlign (DNAStar – Lasergene software) and the ClustalW alignment logarithm. Red boxes indicate important sites inside the sequence, such as phosphorylation sites (red arrows), catalytic sites (CXXC motif) or the putative calcineurin binding motif (box 2). The protein is predicted to be a transmembrane protein with two transmembrane domains, a small cytosolic and a large ER loop. Prediction was performed with the programs TMHMM (http://www.cbs.dtu.dk/services/TMHMM/) and SACS MEMSAT2 (Jones et al., 1994). Phosphorylation sites were predicted by KinasePhos (Huang et al., 2005). **(B)** Diagnostic PCR shows the deletion of *bcpdi1* using the primers: 5′ homologous integration 32 + 43, 3′ homologous integration: 33 + 42, WT: 23 + 24 (see **Supplementary Table S2**). **(C)** Southern blot analysis of three independent Δ*bcpdi1* mutants illustrates the absence of any ectopic integrations of the deletion cassette. Genomic DNA was digested with *Pvu*I; detection took place with the 5′ flank (dotted line). For the WT a fragment size of 5.2 kb was expected while the fragments of the mutants are expected at a size of 3.5 kb. The *bcpdi1* gene was replaced by a hygromycin resistance cassette by homologous recombination via the 5′ and 3′ flank of the gene.

**FIGURE S2 |** BcPdi1 interacts with different proteins of various signaling cascades in yeast-two hybrid assays. Proteins of interest were co-transformed in to *S. cerevisiae* NMY51 and plated on selective media (SD^-^leu^-^trp)/(SD^-^leu^-^his^-^trp^-^ade). For drop tests, the yeast strains were cultivated in YPAD medium, starved out in 1 M sorbitol for 5–6 h and dropped on to selective media (SD^-^leu^-^his^-^trp^-^ade + X-gal + 3AT). Each time dilutions were prepared in advance (1:10 up to 1:1000). For negative controls, the respective empty vector was co-transformed. For the positive control, the vecto constructs pOST_NubI was co-transformed.

**TABLE S1 |** Strains used in this work.

**TABLE S2 |** Oligonucleotides used in this work.

## References

[B1] Armentrout V. M. Downer A. J. (1987). Infection cushion development by *Rhizoctonia solarti* on cotton. *Phytopath* 77:619 10.1094/Phyto-77-619

[B2] Baksh S. Burns K. Andrin C. Michalak M. (1995). Interaction of calreticulin with protein disulfide isomerase. *J. Biol. Chem.* 270 31338–31344. 10.1074/jbc.270.52.31338 8537405

[B3] Beckman K. B. Ames B. N. (1998). The free radical theory of aging matures. *Physiol. Rev.* 78 547–581. 9562038 10.1152/physrev.1998.78.2.547

[B4] Bedard K. Krause K. H. (2007). The NOX family of ROS-generating NADPH oxidases: physiology and pathophysiology. *Physiol. Rev.* 87 245–313. 10.1152/physrev.00044.2005 17237347

[B5] Broome J. C. English J. T. Marois J. J. Latorre B. A. Aviles J. C. (1995). Development of an infection model for *Botrytis* bunch rot of grapes based on wetness duration and temperature. *Phytopathology* 85 97–102. 10.1094/Phyto-85-97

[B6] Buettner P. Koch F. Voigt K. Quidde T. Risch S. Blaich R. (1994). Variations in ploidy among isolates of *Botrytis cinerea*: implications for genetic and molecular analyses. *Curr. Genet.* 25 445–450. 10.1007/BF00351784 8082191

[B7] Cenis J. L. (1992). Rapid extraction of fungal DNA for PCR amplification. *Nucleic Acids Res.* 20:2380 10.1093/nar/20.9.2380 PMC312363 1594460

[B8] Church G. M. Gilbert W. (1984). Genomic sequencing. *Proc. Natl. Acad. Sci. U.S.A.* 81 1991–1995. 10.1073/pnas.81.7.1991 6326095 PMC345422

[B9] Colot H. V. Park G. Turner G. E. Ringelberg C. Crew C. M. Litvinkova L. (2006). A high-throughput gene knockout procedure for *Neurospora* reveals functions for multiple transcription factors. *Proc. Natl. Acad. Sci. U.S.A.* 103 10352–10357. 10.1073/pnas.0601456103 16801547 PMC1482798

[B10] Corbett E. F. Michalak M. (2000). Calcium, a signaling molecule in the endoplasmic reticulum? *Trends Biochem. Sci.* 25 307–311. 10871879 10.1016/s0968-0004(00)01588-7

[B11] de A Paes A. M. Verissimo-Filho S. Guimaraes L. L. Silva A. C. Takiuti J. T. Santos C. X. (2011). Protein disulfide isomerase redox-dependent association with p47(phox): evidence for an organizer role in leukocyte NADPH oxidase activation. *J. Leukoc. Biol.* 90 799–810. 10.1189/jlb.0610324 21791598

[B12] Doehlemann G. Berndt P. Hahn M. (2006). Different signalling pathways involving a Gα protein, cAMP and a MAP kinase control germination of *Botrytis cinerea* conidia. *Mol. Microbiol.* 59 821–835. 10.1111/j.1365-2958.2005.04991.x 16420354

[B13] Elad Y. Pertot I. Cores-Prado A. M. Stewart A. (2016). “Plant hosts of *Botrytis* spp,” in *Botrytis – The Fungus, The Pathogen and its Management in Agricultural Systems*, eds Fillinger S. Elad Y. (Berlin: Springer).

[B14] Farquhar R. Honey N. Murant S. J. Bossier P. Schultz L. Montgomery D. (1991). Protein disulfide isomerase is essential for viability in *Saccharomyces cerevisiae*. *Gene* 108 81–89. 10.1016/0378-1119(91)90490-3 1761235

[B15] Fernandes D. C. Manoel A. H. O. Wosniak J. Laurindo F. R. (2009). Protein disulfide isomerase overexpression in vascular smooth muscle cells induces spontaneous preemptive NADPH oxidase activation and Nox1 mRNA expression: effects of nitrosothiol exposure. *Arch. Biochem.* 484 197–204. 10.1016/j.abb.2009.01.022 19402212

[B16] Gietz R. D. Woods R. A. (2002). Transformation of yeast by lithium acetate/single-stranded carrier DNA/polyethylene glycol method. *Methods Enzymol.* 350 87–96. 10.1016/S0076-6879(02)50957-5 12073338

[B17] Gomi M. Akazawa F. Mitaku S. (2000). SOSUIsignal: software system for prediction of signal peptide and membrane protein. *Genome Inform.* 11 414–415.

[B18] Govrin E. M. Levine A. (2000). The hypersensitive response facilitates plant infection by the necrotrophic pathogen *Botrytis cinerea*. *Curr. Biol.* 10 751–757. 10.1016/S0960-9822(00)00560-1 10898976

[B19] Graf A. Gasser B. Dragosits M. Sauer M. Leparc G. G. Tüchler T. (2008). Novel insights into the unfolded protein response using *Pichia pastoris* specific DNA microarrays. *BMC Genom.* 9:390 10.1186/1471-2164-9-390 PMC2533675 18713468

[B20] Grimm S. (2012). The ER–mitochondria interface: the social network of cell death. *Biochim. Biophys. Acta (BBA)-Mol. Cell Res.* 1823 327–334. 10.1016/j.bbamcr.2011.11.018 22182703

[B21] Gronover C. S. Kasulke D. Tudzynski P. Tudzynski B. (2001). The role of G protein alpha subunits in the infection process of the gray mold fungus *Botrytis cinerea*. *Mol. Plant Microbe. Interact.* 11 1293–1302. 10.1094/MPMI.2001.14.11.1293 11763127

[B22] Haefliger S. Klebig C. Schaubitzer K. Schardt J. Timchenko N. Mueller B. U. (2011). Protein disulfide isomerase blocks CEBPA translation and is up-regulated during the unfolded protein response in AML. *Blood* 117 5931–5940. 10.1182/blood-2010-08-304485 21471526 PMC3293752

[B23] Harren K. Schumacher J. Tudzynski B. (2012). The Ca^2+^/calcineurin-dependent signaling pathway in the gray mold *Botrytis cinerea*: the role of calcipressin in modulating calcineurin activity. *PLoS ONE* 7:e41761 10.1371/journal.pone.0041761 PMC3402410 22844520

[B24] Heimel K. Freitag J. Hampel M. Ast J. Bolker M. Kamper J. (2013). Crosstalk between the unfolded protein response and pathways that regulate pathogenic development in *Ustilago maydis*. *Plant Cell* 25 4262–4277. 10.1105/tpc.113.115899 24179126 PMC3877826

[B25] Heller J. Meyer A. J. Tudzynski P. (2012). Redox-sensitive GFP2: use of the genetically encoded biosensor of the redox status in the filamentous fungus *Botrytis cinerea*. *Mol. Plant Pathol.* 8 935–947. 10.1111/j.1364-3703.2012.00802.x PMC6638776 22524254

[B26] Heller J. Tudzynski P. (2011). Reactive oxygen species in phytopathogenic fungi: signaling, development, and disease. *Annu. Rev. Phytopathol.* 49 369–390. 10.1146/annurev-phyto-072910-095355 21568704

[B27] Huang H. D. Lee T. Y. Tzeng S. W. Horng J. T. (2005). KinasePhos: a web tool for identifying protein kinase-specific phosphorylation sites. *Nucleic Acids Res.* 33 W226–W229. 10.1093/nar/gki471 15980458 PMC1160232

[B28] Janiszewski M. Lopes L. R. Carmo A. O. Pedro M. A. Brandes R. P. Santos C. X. (2005). Regulation of NAD(P)H oxidase by associated protein disulfide isomerase in vascular smooth muscle cells. *J. Biol. Chem.* 280 40813–40819. 10.1074/jbc.M509255200 16150729

[B29] Jones D. T. Taylor W. R. Thornton J. M. (1994). A model recognition approach to the prediction of all-helical membrane protein structure and topology. *Biochemistry* 33 3038–3049. 10.1021/bi00176a037 8130217

[B30] Jung K.-W. Kang H. A. Bahn Y.-S. (2013). Essential roles of the Kar2/BiP molecular chaperone downstream of the UPR pathway in *Cryptococcus neoformans*. *PloS ONE* 8:e58956 10.1371/journal.pone.0058956 PMC3590199 23484059

[B31] Kersteen E. A. Raines R. T. (2003). Catalysis of protein folding by protein disulfide isomerase and small-molecule mimics. *Antioxid. Redox Signal.* 5 413–424. 10.1089/152308603768295159 13678529 PMC2814249

[B32] Kim J. Mayfield S. P. (1997). Protein disulfide isomerase as a regulator of chloroplast translational activation. *Science (New York, N.Y.)* 278 1954–1957. 10.1126/science.278.5345.1954 9395399

[B33] Klimpel A. Gronover C. S. Williamson B. Stewart J. A. Tudzynski B. (2002). The adenylate cyclase (BAC) in *Botrytis cinerea* is required for full pathogenicity. *Mol. Plant Pathol.* 3 439–450. 10.1046/j.1364-3703.2002.00137.x 20569351

[B34] Ko H. S. Uehara T. Nomura Y. (2002). Role of ubiquilin associated with protein-disulfide isomerase in the endoplasmic reticulum in stress-induced apoptotic cell death. *J. Biol. Chem.* 277 35386–35392. 10.1074/jbc.M203412200 12095988

[B35] Koch G. L. (1990). The endoplasmic reticulum and calcium storage. *Bioessays* 12 527–531. 10.1002/bies.950121105 2085319

[B36] Kornmann B. Walter P. (2010). ERMES-mediated ER-mitochondria contacts: molecular hubs for the regulation of mitochondrial biology. *J. Cell Sci.* 123(Pt 9), 1389–1393. 10.1242/jcs.058636 20410371 PMC2858017

[B37] Lambeth J. D. (2004). NOX enzymes and the biology of reactive oxygen. *Nat. Rev. Immunol.* 4 181–189. 10.1038/nri1312 15039755

[B38] Laurindo F. R. Araujo T. L. Abrahao T. B. (2014). Nox NADPH oxidases and the endoplasmic reticulum. *Antioxid. Redox Signal.* 20 2755–2775. 10.1089/ars.2013.5605 24386930 PMC4026305

[B39] Laurindo F. R. Fernandes D. C. Amanso A. M. Lopes L. R. Santos C. X. C. (2008). Novel role of protein disulfide isomerase in the regulation of NADPH oxidase activity: pathophysiological implications in vascular diseases. *Antioxid. Redox Signal.* 10 1101–1114. 10.1089/ars.2007.2011 18373437

[B40] Li C. P. Larkins B. A. (1996). Expression of protein disulfide isomerase is elevated in the endosperm of the maize floury-2 mutant. *Plant Mol. Biol.* 30 873–882. 10.1007/BF00020800 8639747

[B41] Lucero H. A. Lebeche D. Kaminer B. (1998). ER calcistorin/protein-disulfide isomerase acts as a calcium storage protein in the endoplasmic reticulum of a living cell. Comparison with calreticulin and calsequestrin. *J. Biol. Chem.* 273 9857–9863. 10.1074/jbc.273.16.9857 9545326

[B42] Madamanchi N. R. Runge M. S. (2010). NADPH oxidases and atherosclerosis: unraveling the details. *Am. J. Physiol. Heart Circ. Physiol.* 298 H1–H2. 10.1152/ajpheart.01020.2009 19897705 PMC3774417

[B43] Marchi S. Patergnani S. Pinton P. (2014). The endoplasmic reticulum–mitochondria connection: one touch, multiple functions. *Biochim. Biophys. Acta (BBA)* 1837 461–469. 10.1016/j.bbabio.2013.10.015 24211533

[B44] Marschall R. Siegmund U. Burbank J. Tudzynski P. (2016a). Update on Nox function, site of action and regulation in *Botrytis cinerea*. *Fungal Biol. Biotechnol.* 3:8 10.1186/s40694-016-0026-6 PMC5611593 28955467

[B45] Marschall R. Schumacher J. Siegmund U. Tudzynski P. (2016b). Chasing stress signals – exposure to extracellular stimuli differentially affects the redox state of cell compartments in the wild type and signaling mutants of *Botrytis cinerea*. *Fung. Genet. Biol.* 90 12–22. 10.1016/j.fgb.2016.03.002 26988904

[B46] Marschall R. Tudzynski P. (2016a). BcIqg1, a fungal IQGAP homolog, interacts with NADPH oxidase, MAP kinase and calcium signaling proteins and regulates virulence and development in *Botrytis cinerea*. *Mol. Microbiol.* 101 281–298. 10.1111/mmi.13391 27062300

[B47] Marschall R. Tudzynski P. (2016b). Reactive oxygen species in development and infection processes. *Sem. Cell and Dev. Biol.* 57 138–146. 10.1016/j.semcdb.2016.03.020 27039026

[B48] McIlvaine T. C. (1921). A buffer solution for colorimetric comparison. *J. Biol Chem.* 49 183–186.

[B49] Merksamer P. I. Trusina A. Papa F. R. (2008). Real-time redox measurements during endoplasmic reticulum stress reveal interlinked protein folding functions. *Cell* 135 933–947. 10.1016/j.cell.2008.10.011 19026441 PMC2739138

[B50] Mezghrani A. Fassio A. Benham A. Simmen T. Braakman I. Sitia R. (2001). Manipulation of oxidative protein folding and PDI redox state in mammalian cells. *EMBO J.* 20 6288–6296. 10.1093/emboj/20.22.6288 11707400 PMC125306

[B51] Montenegro-Montero A. Goity A. Larrondo L. F. (2015). The bZIP transcription factor HAC-1 is involved in the unfolded protein response and is necessary for growth on cellulose in *Neurospora crassa*. *PLoS ONE* 10:e0131415 10.1371/journal.pone.0131415 PMC4488935 26132395

[B52] Mori K. Ogawa N. Kawahara T. Yanagi H. Yura T. (1998). Palindrome with spacer of one nucleotide is characteristic of the cis-acting unfolded protein response element in *Saccharomyces cerevisiae*. *J. Biol. Chem.* 273 9912–9920. 10.1074/jbc.273.16.9912 9545334

[B53] Mori K. Sant A. Kohno K. Normington K. Gething M. J. Sambrook J. F. (1992). A 22 bp cis-acting element is necessary and sufficient for the induction of the yeast KAR2 (BiP) gene by unfolded proteins. *EMBO J.* 11 2583–2593. 1628622 10.1002/j.1460-2075.1992.tb05323.x PMC556733

[B54] Ngiam C. Jeenes D. J. Punt P. J. Van Den Hondel C. A. Archer D. B. (2000). Characterization of a foldase, protein disulfide isomerase A, in the protein secretory pathway of *Aspergillus niger*. *Appl. Environ. Microbiol.* 66 775–782. 10.1128/AEM.66.2.775-782.2000 10653750 PMC91895

[B55] Perri E. R. Thomas C. J. Parakh S. Spencer D. M. Atkin J. D. (2016). The unfolded protein response and the role of protein disulfide isomerase in neurodegeneration. *Front. Cell Dev. Biol.* 3:80 10.3389/fcell.2015.00080 PMC4705227 26779479

[B56] Pontecorvo G. Roper J. A. Hemmons L. M. MacDonald K. D. Bufton A. W. (1953). The genetics of *Aspergillus nidulans*. *Adv. Genet.* 5 141–238. 10.1016/s0065-2660(08)60408-3 13040135

[B57] Roca M. G. Weichert M. Siegmund U. Tudzynski P. Fleissner A. (2012). Germling fusion via conidial anastomosis tubes in the grey mold *Botrytis cinerea* requires NADPH oxidase activity. *Fungal Biol.* 116 379–387. 10.1016/j.funbio.2011.12.007 22385620

[B58] Roy K. Wu Y. Meitzler J. L. Juhasz A. Liu H. Jiang G. (2015). NADPH oxidases and cancer. *Clin. Sci.* 128 863–875. 10.1042/CS20140542 25818486

[B59] Saloheimo M. Lund M. Penttilä M. E. (1999). The protein disulphide isomerase gene of the fungus *Trichoderma reesei* is induced by endoplasmic reticulum stress and regulated by the carbon source. *Mol. Gen. Genet. MGG* 262 35–45. 10.1007/s004380051057 10503534

[B60] Schumacher J. (2012). Tools for *Botrytis cinerea:* new expression vectors make the gray mold fungus more accessible to cell biology approaches. *Fungal Genet. Biol.* 49 483–497. 10.1016/j.fgb.2012.03.005 22503771

[B61] Schumacher J. Pradier J. M. Simon A. Traeger S. Moraga J. Collado I. G. (2012). Natural variation in the VELVET gene *bcvel1* affects virulence and light-dependent differentiation in *Botrytis cinerea*. *PLoS ONE* 7:e47840 10.1371/journal.pone.0047840 PMC3485325 23118899

[B62] Schwarzlaender M. Fricker M. D. Mueller C. Marty L. Brach T. Novak J. (2008). Confocal imaging of glutathione redox potential in living plant cells. *J. Microsc.* 231 299–316. 10.1111/j.1365-2818.2008.02030.x 18778428

[B63] Segmueller N. Kokkelink L. Giesbert S. Odinius D. van Kan J. Tudzynski P. (2008). NADPH oxidases are involved in differentiation and pathogenicity in *Botrytis cinerea*. *Mol. Plant Microbe Interact.* 21 808–819. 10.1094/MPMI-21-6-0808 18624644

[B64] Siegmund U. Heller J. van Kan J. A. Tudzynski P. (2013). The NADPH oxidase complexes in *Botrytis cinerea*: evidence for a close association with the ER and the tetraspanin Pls1. *PLoS ONE* 8:e55879 10.1371/journal.pone.0055879 PMC3572182 23418468

[B65] Siegmund U. Marschall R. Tudzynski P. (2015). BcNoxD, a putative ER protein, is a new component of the NADPH oxidase complex in *Botrytis cinerea*. *Mol. Microbiol.* 95 988–1005. 10.1111/mmi.12869 25402961

[B66] Sievers F. Wilm A. Dineen D. Gibson T. J. Karplus K. Li W. (2011). Fast, scalable generation of high-quality protein multiple sequence alignments using clustal omega. *Mol. Syst. Biol.* 7:539 10.1038/msb.2011.75 PMC3261699 21988835

[B67] Steinhorst L. Kudla J. (2014). Signaling in cells and organisms—calcium holds the line. *Curr. Opin. Plant Biol.* 22 14–21. 10.1016/j.pbi.2014.08.003 25195171

[B68] Stoica R. De Vos K. J. Paillusson S. Mueller S. Sancho R. M. Lau K. (2014). ER–mitochondria associations are regulated by the VAPB–PTPIP51 interaction and are disrupted by ALS/FTD-associated TDP-43. *Nat. Commun.* 5:3996 10.1038/ncomms4996 PMC4046113 24893131

[B69] Sullivan D. C. Huminiecki L. Moore J. W. Boyle J. J. Poulsom R. Creamer D. (2003). EndoPDI, a novel protein-disulfide isomerase-like protein that is preferentially expressed in endothelial cells acts as a stress survival factor. *J. Biol. Chem.* 278 47079–47088. 10.1074/jbc.M308124200 12963716

[B70] Tan K. K. Epton H. (1973). Effect of light on the growth and sporulation of *Botrytis cinerea*. *Trans. Br. Mycol. Soc.* 61 145–157. 10.3389/fpls.2016.00625

[B71] Tudzynski P. Heller J. Siegmund U. (2012). Reactive oxygen species generation in fungal development and pathogenesis. *Curr. Opin. Microbiol.* 15 653–659. 10.1016/j.mib.2012.10.002 23123514

[B72] Viefhues A. Heller J. Temme N. Tudzynski P. (2014). Redox systems in *Botrytis cinerea*: impact on development and virulence. *Mol. Plant-Microbe. Interact.* 27 858–874. 10.1094/MPMI-01-14-0012-R 24983673

[B73] Wilkinson B. L. Landreth G. E. (2006). The microglial NADPH oxidase complex as a source of oxidative stress in alzheimer’s disease. *J. Neuroinflammation* 3:30 10.1186/1742-2094-3-30 PMC1637099 17094809

[B74] Zeeshan H. M. A. Lee G. H. Kim H. Chae H. (2016). Endoplasmic reticulum stress and associated ROS. *Int. J. Mol. Sci.* 17:327 10.3390/ijms17030327 PMC4813189 26950115

