# Supplementary figures and images for: The Protein Disulfide Isomerase of Botrytis cinerea: An ER Protein Involved in Protein Folding and Redox Homeostasis Influences NADPH Oxidase Signaling Processes
Source: Front Microbiol. 2017 May 29;8:960. doi: 10.3389/fmicb.2017.00960 (PMC5447010; doi:10.3389/fmicb.2017.00960)

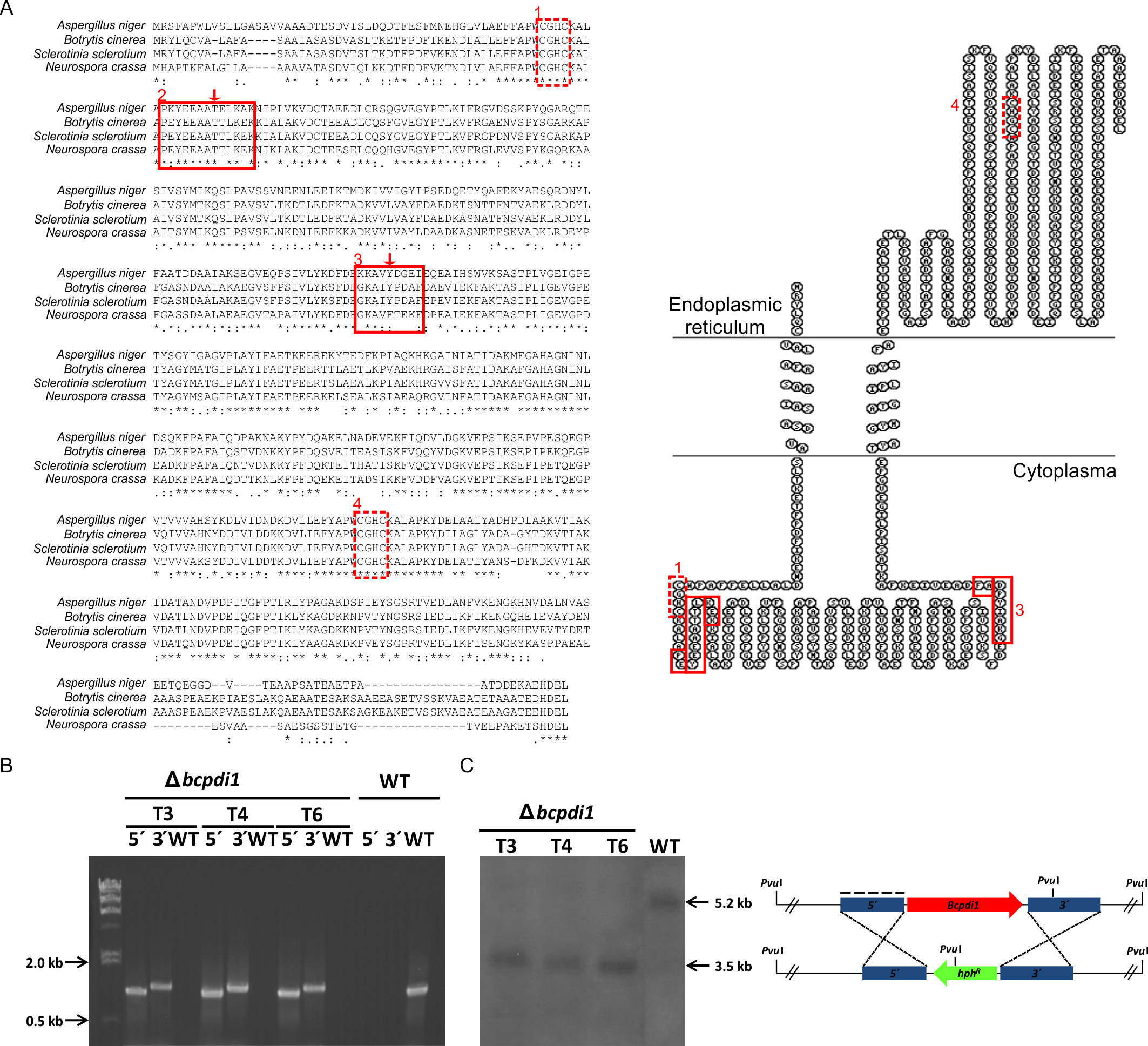

Supplement: Supplementary file 1 [file Image_1.TIF]

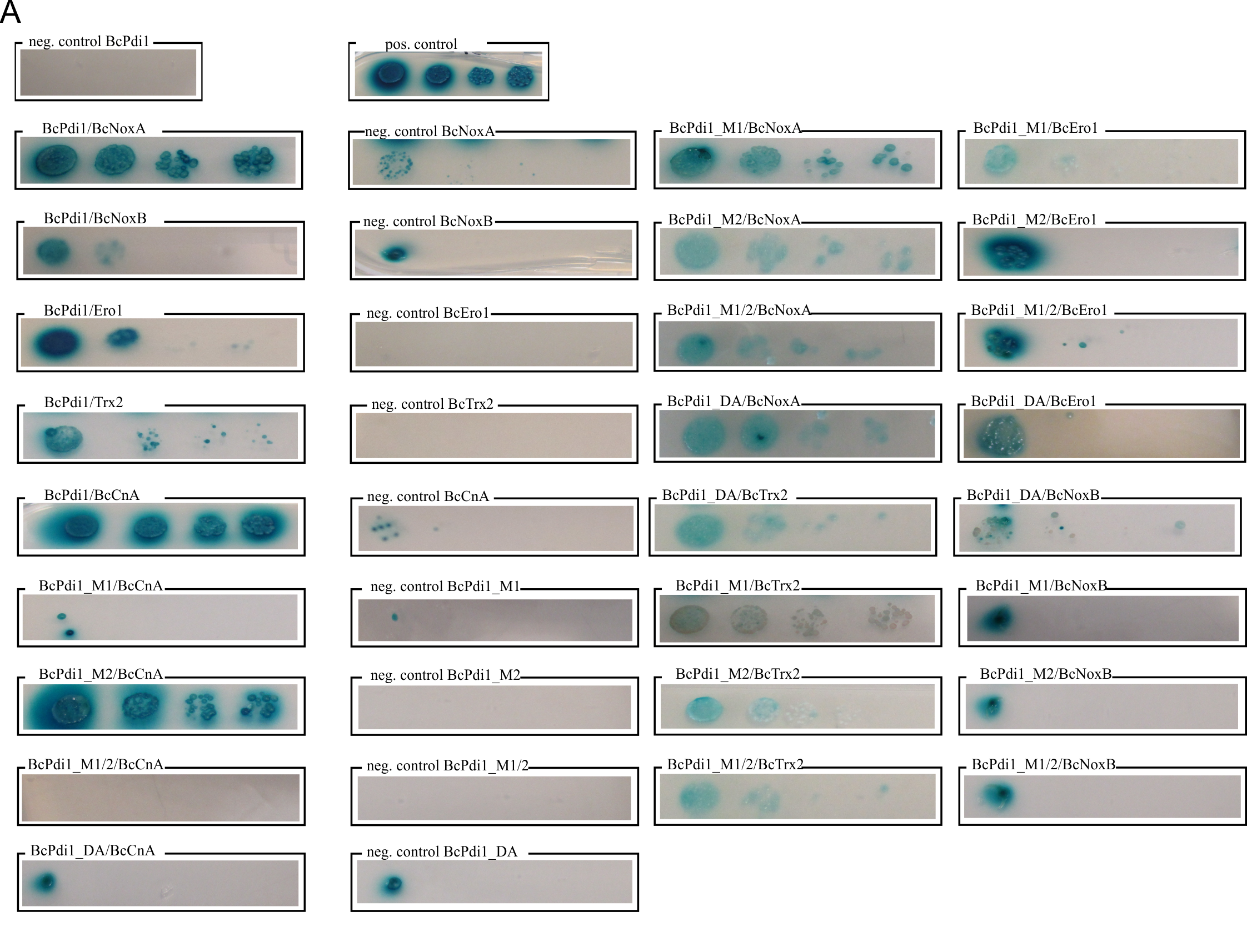

Supplement: Supplementary file 2 [file Image_2.TIF]
